# Untargeted metabolomics revealed urinary metabolic pattern for discriminating prostate cancer from benign prostatic hyperplasia in Chinese participants

**DOI:** 10.3389/fonc.2025.1604169

**Published:** 2025-06-25

**Authors:** Chenye Tang, Xiao Guo, Qian Zou, Xinghao Wang, Jian Sheng, Dan Shen, Chun Sun, Shuo Li, Ruilin Shen

**Affiliations:** ^1^ Department of Urology, The Second Hospital of Jiaxing, Jiaxing, Zhejiang, China; ^2^ Jiaxing University Master Degree Cultivation Base, Zhejiang Chinese Medical University, Hangzhou, Zhejiang, China; ^3^ Department of Science and Education, The Second Hospital of Jiaxing, Jiaxing, Zhejiang, China; ^4^ Zhejiang Key Laboratory of Digital Technology in Medical Diagnostics, Dian Diagnostics Group Co., Ltd., Hangzhou, Zhejiang, China

**Keywords:** prostatic hyperplasia, prostate cancer, urinary metabolomics, discriminant biomarkers, untargeted metabolomics

## Abstract

**Introduction:**

Precise screening and discriminating of prostatic hyperplasia (PH) could avoid unnecessary biopsy and overdiagnosis. However, the metabolic pattern of patients with prostatic hyperplasia in Chinese population is rarely reported.

**Methods:**

Urine samples of Chinese participants with prostate cancer (PCa), benign prostatic hyperplasia (BPH) and non-prostate diseases (NPD) were detected with four ultra-performance liquid chromatography/tandem mass spectrometric (UPLC-MS/MS) methods to profile the metabolic disturbance.

**Results:**

In patients with PH, the most significant dysregulation was observed in metabolites categorized as lipid or amino acid, especially those involved in histidine metabolism, purine metabolism, tryptophan metabolism and tyrosine metabolism. For discrimination BPH from PCa, apart from previously reported metabolites related to phospholipid metabolism or tryptophan metabolism, metabolites of dipeptides and androgenic steroids, such as leucylhydroxyproline and etiocholanolone glucuronide, also exhibited potential to discriminate PCa from BPH.

**Conclusion:**

This study conducts precise detection of urinary metabolomic pattern for patients with benign prostatic hyperplasia or prostate cancer, and could inform their potential application as discriminant biomarkers.

## 1 Introduction

Prostate cancer (PCa) is one of the most common malignancies among men globally, with an incidence rate of 7.3% and a mortality rate of 4.0%, ranking fourth and eighth respectively in global population, according to the latest epidemiological survey in 2022 (1). In China, the burden of PCa is also steadily increasing, with an estimated 315,310 cases projected by 2030 (2). Early screening and accurate diagnosis of PCa are crucial to address the clinical needs and enhance public awareness of the disease.

Guidelines recommended methods for PCa screening (3, 4) mainly include serology tests, imaging examinations and palpation of digital rectal examination (DRE) (4). However, imaging tests could be costly and hardly accessible in primary healthcare facilities. DRE exhibited difficulty for discriminating benign prostatic hyperplasia (BPH) from prostate cancer. The prostate-specific antigen (PSA) test, suffers from low sensitivity and specificity issues leading to either missed diagnosis of poorly differentiated PCa or overdiagnosis due to excessive screening (5, 6). Currently, needle biopsy and surgical pathology remain as gold standard for clinical diagnosis (7), but both are invasive and often complained post-operational discomfort for patients (8). Therefore, identifying non-invasive diagnostic markers holds highlights for enhancing the effectiveness of PCa screening and management.

Efforts of discovering PCa-related markers have been made and recommended (3) based on multiple techniques. For instance, rearrangements of the transmembrane serine protease 2 (TMPRSS2) and erythroblast transformation-specific-related gene (ERG) on chromosome 21q22 have been identified as potential loci for PCa development (9, 10). Urinary exosomal RNA was recommended as auxiliary marker for early screening and adjunctive diagnosis (11, 12). Additionally, perturbations in metabolite levels play a pivotal role in the initiation and progression of PCa (13, 14), and certain organic acid molecules hold potential as biomarkers for PCa screening and differentiation (15, 16). Given that metabolomics reflects downstream physiology changes compared to genome and transcriptome (17), urinary metabolic patterns hold promise for biomarker discovery. However, researches conducted on the Chinese mainland are with limited number according to the research investigation.

In this study, we hypothesized that prostatic hyperplasia lead to unique metabolic changes in the urine of patients, which could exhibit various pattern in different population. Therefore, participants with prostatic hyperplasia or no prostate diseases were recruited from a single center on the Chinese mainland. Urine samples were collected for metabolomics detection and bioinformatic analysis. Four methods of ultra-performance liquid chromatography/tandem mass spectrometry (UPLC-MS/MS) were utilized for precise detection, and specific metabolites of lipid and amino acids were reported dysregulation in prostatic hyperplasia patients, carrying potential to discriminate PCa from BPH.

## 2 Materials and methods

### 2.1 Subject enrollment and eligibility criteria

From January 2022 to December 2022, male participants with an suspicious risk of prostate cancer were enrolled for this study. Basically, the enrolled participants exhibited with an elevated PSA level (≥ 4.0 ng/mL) while community or staff physical checking, or with clinical complaint of symptoms such as increased nocturia and poor urination. The inclusion criteria were as follows: patients with prostatic hyperplasia (PH) by ultrasound were enrolled into PH group, which could contain patients with benign hyperplasia or prostate cancer. For prostate cancer (PCa) group, patients’ needs to be pathologically diagnosed as prostate cancer by aspiration biopsy or (and) surgical pathological examination. For benign prostatic hyperplasia (BPH) group, participants were pathologically diagnosed as benign hyperplasia by surgical examination; for non-prostate disease (NPD) group, subjects were diagnosed without prostate diseases by pathological, imaging and serological tests. Subjects with a history of liver or kidney disorders, digital rectal examination (DRE), or prostate biopsy within one month prior to sample collection would be excluded. Besides, patients with a history of malignant tumor, treatment of urological diseases and other chronic diseases would not be included. Urine samples were collected before pathological examination or DRE and stored at -80°C until metabolomic detection.

This study was approved by the institutional review board of The Second Hospital of Jiaxing (No. JXEY-2022HXHZ040). All participants signed the informed consent form before enrollment.

### 2.2 Urinary untargeted metabolomic detection

Untargeted metabolomic analysis was conducted by Calibra Lab at DIAN Diagnostics (Hangzhou, Zhejiang, China) as previously described (18). Briefly, urine samples were thawed on ice and extracted with methanol at a ratio of 1:4. The resulting mixtures were shaken and precipitated. Four 100 μL aliquots of the supernatant were dried under blowing nitrogen, and subsequently re-dissolved for injection into UPLC-MS/MS systems. To obtain the precise components of samples, untargeted metabolomic detection were conducted with four UPLC-MS/MS methods, all of them based on ACQUITY 2D UPLC (Waters, Milford, MA, USA) plus Q Exactive (QE) hybrid Quadrupole-Orbitrap mass spectrometer (Thermo Fisher Scientific, USA). The QE mass spectrometer was operated at a mass resolution of 35,000, with a scan range of 70–1000 m/z. Detailed platform and mobile solutions of four UPLC-MS/MS methods were consistent with those previously mentioned (19).

### 2.3 Metabolomic data analysis

Raw data of metabolomics were preprocessing and quality control inspection. The ion peaks were extracted based on proprietary in-house IT hardware and software. There would be eight steps of data extraction and analysis, including extraction, smoothing, and noise estimation of extracted ion chromatograms (EIC), selection, integration, screening, and identification of peaks, and batch optimization. It should be noted that the peaks data meeting the following criteria would be excluded: a signal to noise ratio <5, less than 5 scan points, apex intensity less than 3000, and RT width less than 0.02min. Metabolite identification were generated by in-house library searching, as previously published (19). Raw peak area for each metabolite was calculated using area-under-the-curve (AUC).

Raw peak areas were normalized by both urine creatinine and total peak area values. The normalized peak areas were then log-transformed (log2) for further statistical analysis. Missing values in peak area matrix were imputed by using the minimal detection value of the corresponding metabolite among all samples. Statistical analysis included multivariate analysis, such as principal component analysis (PCA) and orthogonal partial least square discriminant analysis (OPLS-DA), to obtain differential metabolites among groups.

### 2.4 Statistics

The statistical differences in clinical characteristics among groups were assessed using the Chi-square test (qualitative variables) and the independent t-test (quantitative variables). For metabolomic data, differential metabolites were found by non-parametric (Kruskal-Wallis test) statistical methods, visualized through volcano plot. Multivariate analysis approaches including PCA and OPLS-DA, were also conducted to discriminate differences among groups. Variable importance in the projection (VIP) were generated in OPLS-DA. Correlation among differential metabolites and clinical features were evaluated by Pearson’s correlation analysis. Enrichment analysis was conducted with MetaboAnalyst (https://www.metaboanalyst.ca/MetaboAnalyst/home.xhtml, updated version Dec. 2023). Result visualization were provided with mixOmics and ggplot2 packages for scatter plot, pheatmap package for heatmaps.

All statistical analyses of were performed with R language (version 4.1.0). *P* < 0.05 was considered with statistical significance.

## 3 Results

### 3.1 Study design and baseline characteristics of enrolled participants

**Figure 1:**
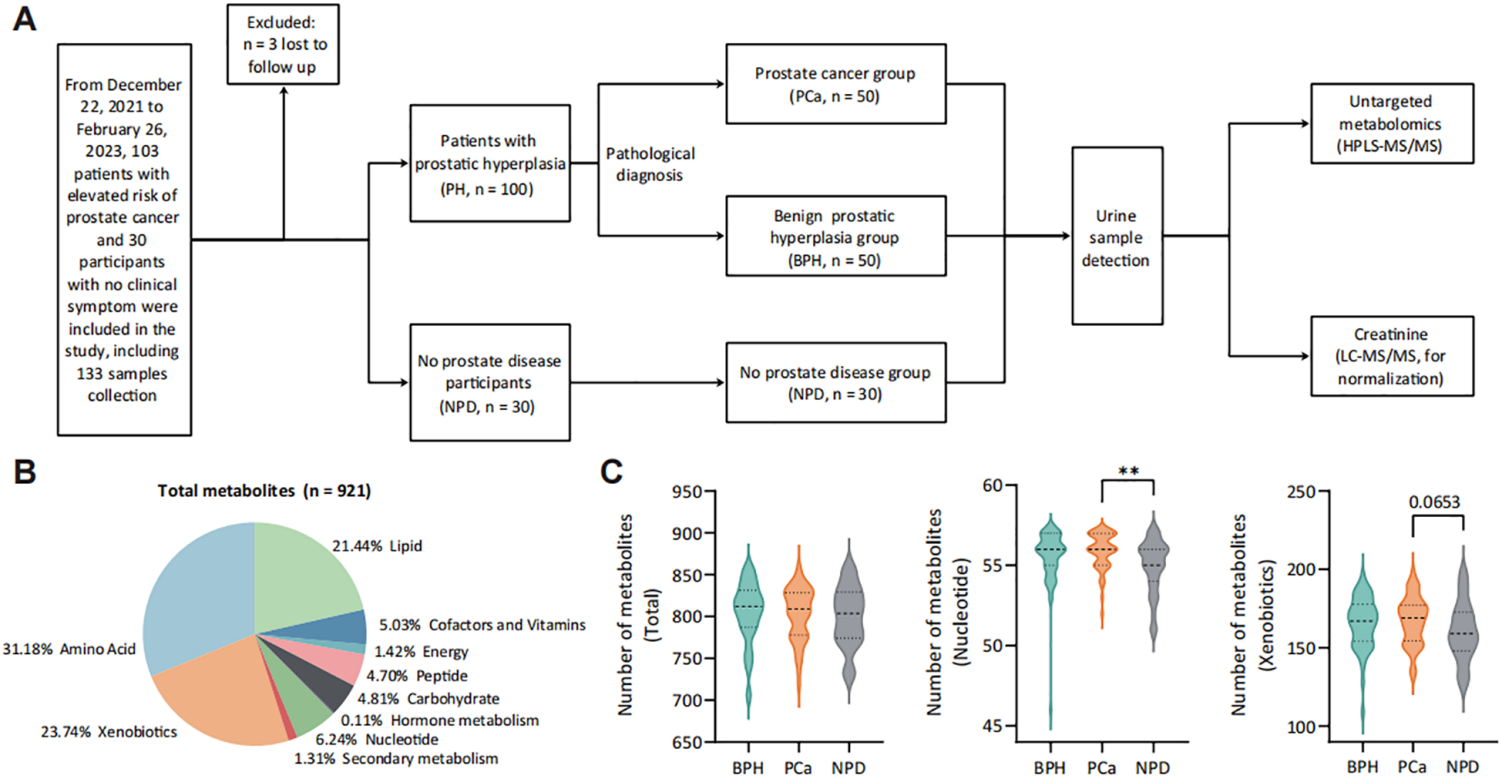
Landscape of study design and urine metabolomic detection. **(A)** Study workflow. **(B)** Categories of detected metabolites from 130 urine samples. **(C)** Number of detected metabolites in each category from each group. **p < 0.01. PH group, patients with prostatic hyperplasia, contains PCa and BPH groups. PCa group, patients pathologically diagnosed with prostate cancer. BPH group, patients pathologically diagnosed with benign prostatic hyperplasia. NPD group, participants without prostate diseases.

A total of 130 urine samples were finally collected for detection of untargeted metabolomics. To reduce the impact of prostate massage or invasive operation, samples were collected prospectively and grouping after pathological, imaging and serological tests (**Figure 1A**). Baseline characteristics of enrolled participants were listed as **Table 1**, and cancer-related features of 50 PCa patients were exhibited in **Table 2**. The PSA level showed significant elevation in patients with prostate nodules, especially in PCa patients (*p* < 0.001).

**Table 1:** Clinical characteristics of enrolled patients (n = 130).

	PCa	BPH	NPD	*p*-value		
	(n = 50)	(n = 50)	(n = 30)	PCa vs BPH	PCa vs NPD	BPH vs NPD
Age/year	71.3 (5.98)	69.9 (8.06)	57.4 (11.2)	0.348	<0.001 ***	<0.001 ***
BMI/kg*m^-2^	24.3 (2.77)	23.0 (2.98)	24.8 (2.63)	0.029 *	0.417	0.008 **
Prostate volume/mL	50.7 (25.5)	29.0 (14.7)	N.D.	<0.001 ***	/	/
PSA/ng*mL^-1^	28.5 (26.9)	06.9 (7.26)	01.8 (1.63)	<0.001 ***	<0.001 ***	<0.001 ***
N.D.	01 (2.0%)	01 (2.0%)	06 (20.0%)			
PSA range/ng*mL^-1^						
< 4.0	01 (2.0%)	23 (46.0%)	21 (70.0%)	<0.001 ***	<0.001 ***	0.003 **
4.0 ~ 10.0	15 (30.0%)	15 (30.0%)	03 (10.0%)			
> 10.0	33 (66.0%)	11 (22.0%)	00 (0%)			
N.D.	01 (2.0%)	01 (2.0%)	06 (20.0%)			

Values of Age, BMI, PSA and Prostate volume were shown as Mean (SD). Others were shown as n (%).

N.D., not detected.

*p < 0.05, **p < 0.01, ***p < 0.001.

**Table 2:** Cancer-related characteristics of PCa patients (n = 50).

	PCa (n = 50)
Gleason_Score	
3 + 3 = 6	9 (18.0%)
3 + 4 = 7	16 (32.0%)
4 + 3 = 7	9 (18.0%)
4 + 4 = 8	5 (10.0%)
4 + 5 = 9	5 (10.0%)
N.D.	6 (12.0%)
TNM_stage	
T2N0M0	39 (78.0%)
T2N1M0	1 (2.0%)
T2N1M1b	1 (2.0%)
T3aN0M0	4 (8.0%)
T3aN1M1b	2 (4.0%)
T3bN0M0	1 (2.0%)
T3bN1M1b	1 (2.0%)
N.D.	1 (2.0%)
Cancer Stage	
I	15 (30.0%)
II	9 (18.0%)
III A	15 (30.0%)
III B	5 (10.0%)
IV A	1 (2.0%)
IV B	4 (8.0%)
N.D.	1 (2.0%)
T_stage	
2	41 (82.0%)
3a	6 (12.0%)
3b	2 (4.0%)
N.D.	1 (2.0%)
N_stage	
0	44 (88.0%)
1	5 (10.0%)
Missing	1 (2.0%)
M_stage	
0	45 (90.0%)
1b	4 (8.0%)
N.D.	1 (2.0%)

Values were shown as n (%).

N.D., not detected.

### 3.2 Metabolomic landscape and differential metabolic pattern of patients with prostatic hyperplasia

Following urine metabolomic detection, a total of 921 metabolites were identified with UPLC-MS/MS (**Figure 1B**), with the majority being metabolites of amino acid (31.18%), xenobiotics (23.74%) and lipid (21.44%). Patients with prostate nodules exhibited an increased number of detected urinary metabolites (**Supplementary Figure S1**), categorized into nucleotide (*p* < 0.05), secondary metabolism (*p* = 0.061) and xenobiotics (*p* = 0.067). For prostate cancer patients, more varieties of urinary nucleotide were detected compared with no prostate disease (NPD) participants (**Figure 1C**).

**Figure 2:**
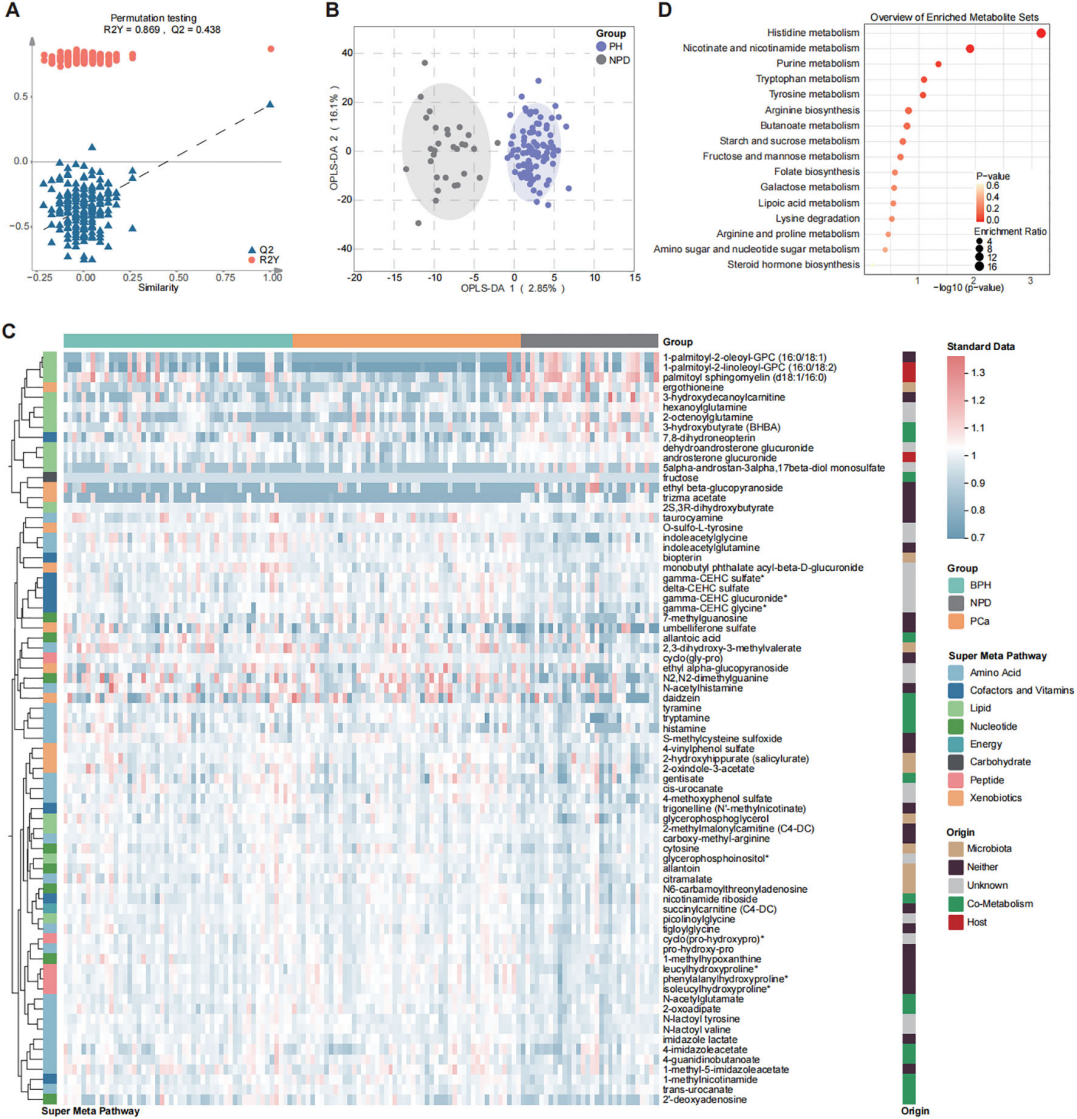
Differential distribution of urine metabolomic profile between PH and NPD by OPLS-DA model. **(A)** Model parameters. **(B)** Plot of structural separation. **(C)** Heatmap of 75 differential metabolites selected with OPLS-DA (VIP > 1.7). **(D)** Enrichment analysis of 75 differential metabolites by using KEGG.

To identify the changing pattern of patients with prostatic hyperplasia (PH), OPLS-DA was performed between PH and NPD groups (**Figures 2A, B**) and differential metabolites were illustrated in **Figure 2C** (OPLS-DA VIP > 1.7). There were 16/75 decreased and 59/75 increased metabolites for patients with prostatic hyperplasia (**Supplementary Figure S2**). Decreased metabolites were mainly categorized with lipids, and increased metabolites principally included amino acids, xenobiotics, and cofactors and vitamins. Results of Kyoto Encyclopedia of Genes and Genomes (KEGG) enrichment analysis exhibited significantly dysregulated pathways related to amino acid metabolism, including histidine, tryptophan and tyrosine metabolism, in addition to purine metabolism. (**Figure 2D**). Purine metabolism supplies crucial building blocks for DNA and RNA synthesis, as well as energy and cofactors that promote cell survival and proliferation. Moreover, purine and amino acid metabolisms are tightly linked, as several amino acids serve as substrates necessary for the purine *de novo* biosynthetic pathway, especially in rapidly proliferating cancer cells (20, 21). Besides, metabolites related to nicotinate and nicotinamide metabolism were also significantly enriched in PH patients.

### 3.3 Key metabolomics to discriminate PCa from BPH patients

**Figure 3:**
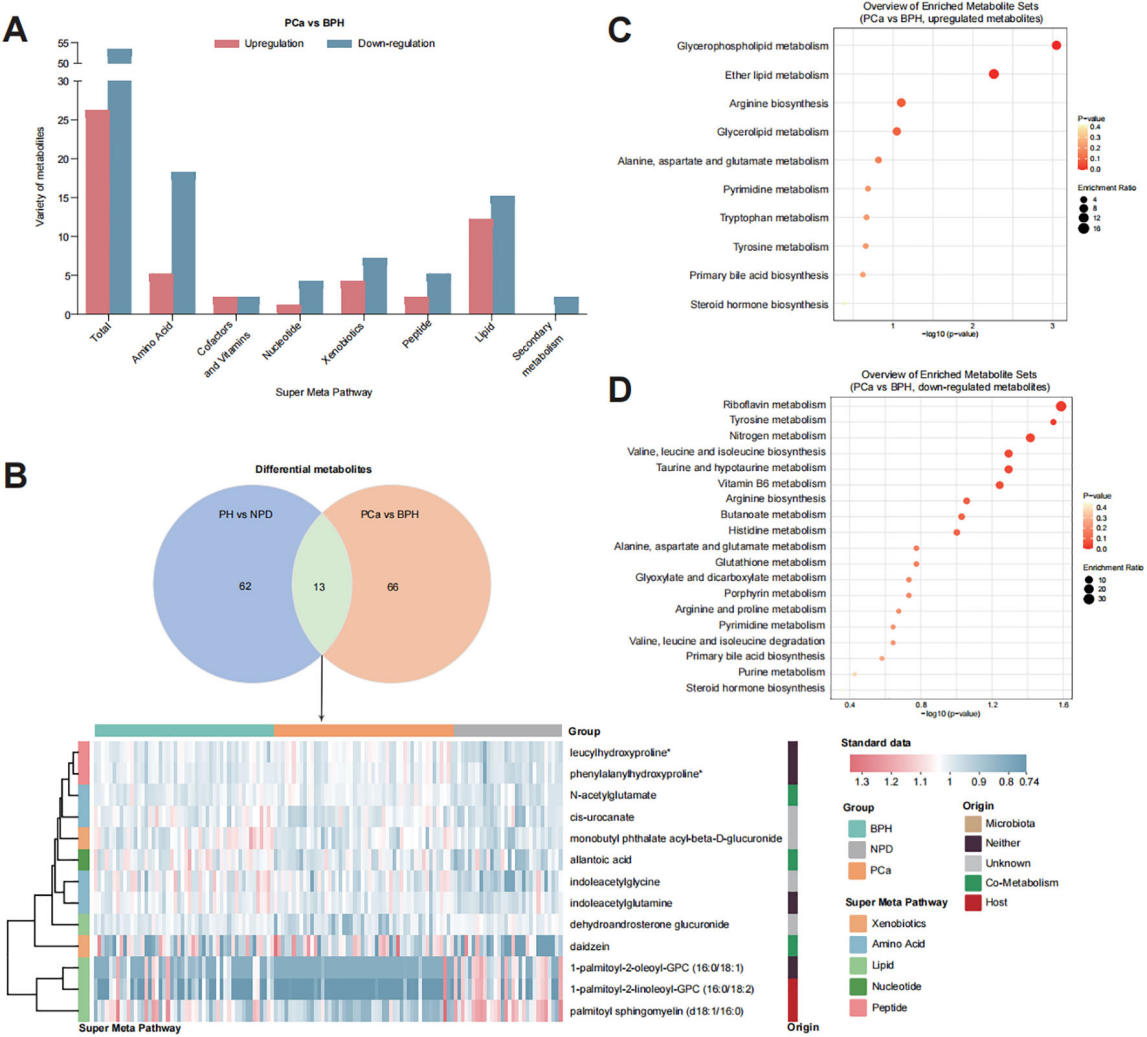
Distribution of key metabolites to discriminate PCa from BPH. **(A)** Bar plot of detected varieties of key metabolites in each category. **(B)** Metabolites contributed both for discriminating PH from NPD and PCa from BPH. **(C)** Enrichment analysis of 26 upregulated metabolites in the urine of PCa group. **(D)** Enrichment analysis of 53 down-regulated metabolites in the urine of PCa patients.

With the purpose of confirming the key differential metabolites between patients with benign prostatic hyperplasia and those with prostate cancer, both univariate and multivariate statistical tests were utilized (**Figure 3**). Results of volcano plot and OPLS-DA model revealed significant differences between BPH and PCa groups. Compared with BPH group, there were 26 increased and 53 decreased urinary metabolites in the PCa group (**Supplementary Figure S3A**).

The main differential metabolites (OPLS-DA VIP > 1.7) were categorized into two major groups: lipid (27 out of 79) and amino acid (23 out of 79). Among these, 13 metabolites were identified to discriminate PN and NPD (**Supplementary Figure S3B**), highlighting their strong suggestive of prostate cancer. KEGG enrichment analysis indicated that the upregulated metabolites were associated with pathways of glycerophospholipid metabolism, ether lipid metabolism and arginine biosynthesis (**Supplementary Figure S3C**), and the down-regulated metabolites were related to pathways of riboflavin metabolism, tyrosine metabolism, nitrogen metabolism, valine, leucine and isoleucine biosynthesis, taurine and hypotaurine metabolism, and vitamin B6 metabolism (**Supplementary Figure S3D**).

### 3.4 Key metabolites significantly correlated with PCa-related features

**Figure 4:**
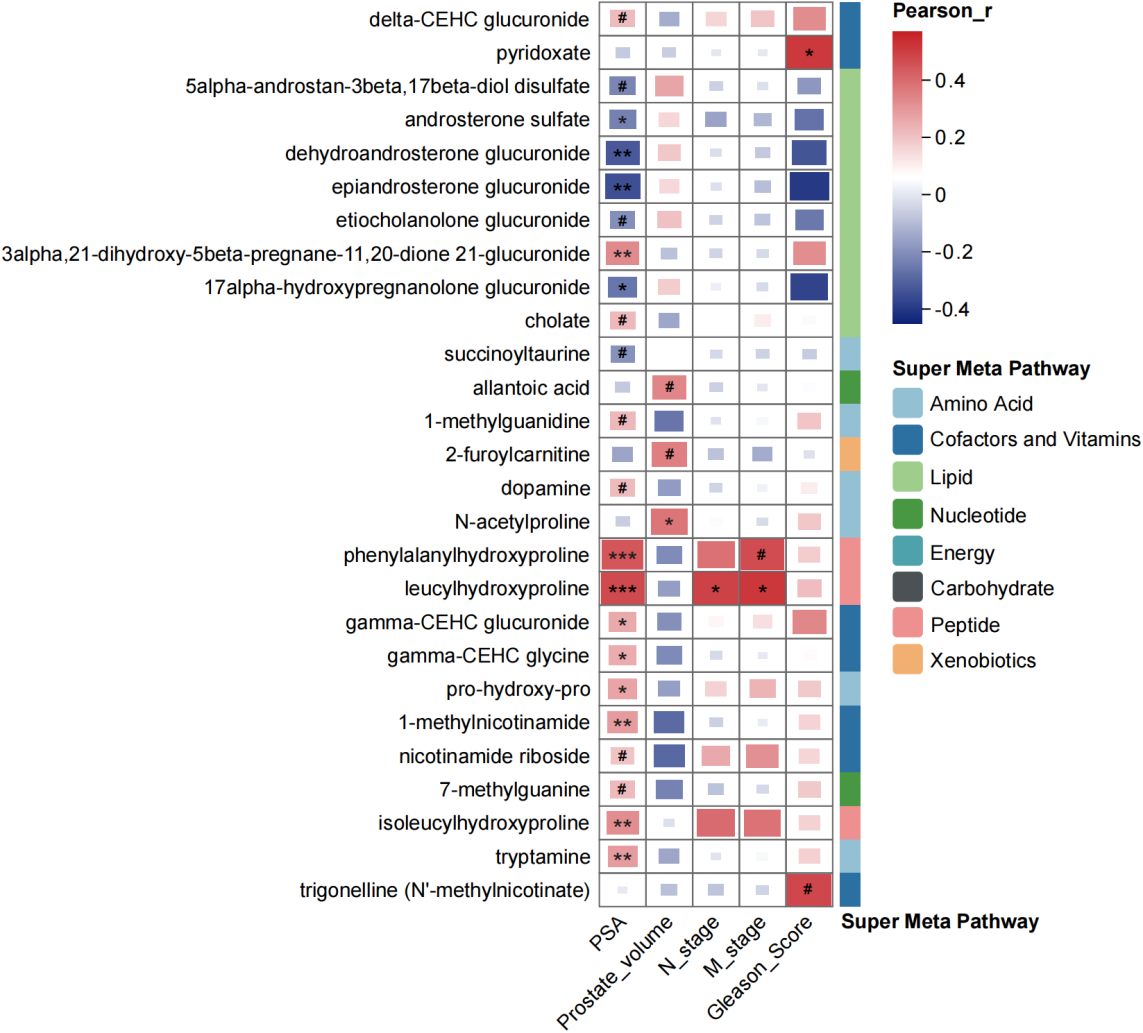
Correlation coefficients among metabolites and clinical characteristics of participants by using Pearson’s correlation analysis. #*p* < 0.1, *p < 0.05, **p < 0.01, ***p < 0.001.

We further conducted analysis to explore the correlation between the selected key metabolites and prostate cancer-related clinical features, such as PSA level, prostate volume and Gleason score, by Pearson’s correlation analysis (**Figure 4**). Among the 79 key metabolites, 27 exhibited significant correlations with clinical features. Notably, several lipid metabolites, such as androsterone sulfate, dehydroandrosterone glucuronide, epiandrosterone glucuronide, ethiocholanolone glucuronide, 17alpha-hydroxypregnanolone glucuronide, showed negative correlations with PSA levels. What was more, certain urinary peptides, such as phenylalanylhydroxyproline, leucylhydroxyproline and isoleucylhydroxyproline, urinary cofactors and vitamins, such as CEHC clucuronide, CEHC glycine, and 1-methylnicotinamide, demonstrated significant positive correlations with PSA levels.

## 4 Discussion

Metabolomics has gained widespread application in biomarker research due to its ability of direct reflection of interactions between genes and the environment, compared with other omics approaches. While there have been advancements in metabolomics studies related to PCa, limited studies have focused on the profiling of urine metabolites in the Chinese PCa population, which might exhibit distinct metabolic features and could be crucial for the discovery of appropriate biomarkers. In this study, Chinese participants with no prostate diseases, benign prostatic hyperplasia, or prostate cancer were recruited, and differential metabolic profiles between PH and NPD, PCa and BPH were featured, by using untargeted metabolomic detection based on four UPLC-MS/MS methods.

Discriminate analysis of urine metabolites between PH and NPD showed that, in the urine of PH patients, lipid metabolites were significantly decreased while amino acid metabolites were increased, significantly (**Figure 2**, **Supplementary Figure S2**). Amino acid metabolites, especially those related to histidine metabolism, purine metabolism, tryptophan metabolism and tyrosine metabolism, have previously been reported related to kidney dysfunction (22), urological carcinoma (23, 24), and even progression of prostate cancer (25). Histidine metabolism has been found to be upregulated in men with T2 prostate cancer (26), and been significantly inhibited after carbon ion radiotherapy (27), which corroborates the decreased levels observed in PH patients in this study. Purine metabolism has been extensively reported associated with urological dysfunction, including diabetic nephropathy (22), aging-induced kidney dysfunction (28), and bladder cancer (29). In this study, we further raised the awareness of its relevance to prostatic hyperplasia, with the elevation of allantoic acid, allantoin, N2,N2-dimethylguanine, and 2’-deoxyadenosine as the main features. Further exploration about the progression of prostatic hyperplasia and even urological cancer could focus on these pronounced metabolic pathways.

After focusing on the dysregulated pathways between PH and NPD, our points of attention concentrated on the key metabolites to discriminate benign hyperplasia from prostate cancer (**Figure 3**, **Supplementary Figure S3**). A total of 79 metabolites were selected by OPLS-DA model for discrimination, with 26 of them being elevated in the urine of PCa patients. Metabolites related to phospholipid metabolism, including glycerophosphorylcholine (GPC) and glycerophosphoethanolamine, were observed to decrease in the castration-resistant prostate cancer cells following treatment with androgen receptor (AR) activation inhibitors (30). Additionally, the level of tetrahydrocortisone has been reported to statistically decrease with AR antagonist treatment, and might serve as a partial pharmacodynamic signal of enzalutamide (31). These findings align with the relatively elevated levels observed in our study among PCa patients, compared with BPH group. Prostate is one of the hormone-sensitive tissues (32), and dysregulation and malfunction of androgenic steroids could result in prostate disorders (33). Many publications have reported the activation of androgen receptor (AR) signaling triggered the progression of PCa. Bui et al. proved that cortisol metabolites derived from gut microbiota could enhance the migration of PCa cells and activate AR and AR-related genes (34). And the expression of AR was significantly connected with PSA expression (35). In this study, urinary androgenic steroids, such as epiandrosterone glucuronide, etiocholanolone glucuronide, androsterone sulfate, and dehydroandrosterone glucuronide, were significantly correlated with serum PSA level (**Figure 4**), and also exhibited distinctive potential to differentiate BPH from PCa (**Supplementary Table S1**). Those results further support the androgenic steroids and AR signaling could regulate the progression of PCa. And those metabolites have not been proposed, to our knowledge.

Apart from those molecules which have been previously published in other sample types, this study identified several metabolites rarely reported associated with prostate cancer in the existing literature. Serum dipeptides have been reported linked to an elevated risk of lethal prostate cancer (36). In this study, we found that phenylalanylhydroxyproline and leucylhydroxyproline play a significant role in discriminating both PH from NPD and BPH from PCa (**Supplementary Figure S3**), and the relative abundances patterns are consistent with the progression from hyperplasia to malignancy. Publications about leucylhydroxyproline (LHP) found that serum LHP be candidate therapeutic markers of zoledronic acid in rats (37), and be associated with developmental dysplasia of the hip (38). Phenylalanylhydroxyproline was reported as active compound to stimulate the production of articular cartilage matrix (39). However, these dipeptides were firstly reported to be connected with prostate cancer, to our knowledgement. In this case, a few references could be explanation for our discovery. We suppose that elevated depeptides suggest the connection between collagen breakdown (40) and PCa incidence. Furthermore, nucleotide metabolites, such as 2’-O-methyluridine, also conducted potential as urinary biomarker for discrimination (**Supplementary Table S1**, AUC = 0.760). It suggested that with the precise detection of multiple UPLC-MS/MS systems, various components of samples could be quantified and exhibited potential for diagnosis.

Finally, this study has several limitations which should be acknowledged. First, while the sample size is adequate for discovery phase, the scale could be further expanded to better evaluate the diagnostic performance as potential biomarkers. Second, in order to align with the broader application of clinical scenario, we chose random urine samples, instead of spot urine. Third, significant differences of age and BMI were observed in the baseline clinical features. To avoid the disturbances, we analyzed the correlation between the differential metabolites and clinical features, and no significance was calculated statistically (data was not shown). Further investigations could concentrate on validating the diagnostic performances with larger sample scale and minimizing potential disturbances.

## 5 Conclusion

Metabolomics has \proven to be well utilized for pattern-feature description and biomarker discovery, with precise detection and specific population be crucial for advancing translational medicine. In this study, we applied four UPLC-MS/MS methods to detect the precise metabolic pattern of PCa, BPH and NPD patients, in the urine samples of Chinese population. Notably, specific metabolites, including certain androgenic steroids and depeptides, have rarely been reported, to our knowledge. However, these metabolites demonstrated potential to discriminant prostatic hyperplasia, especially from prostate cancer, in Chinese patients. Further studies could be conducted with larger sample size and more precise detected platforms to explore the true performances of these potential biomarkers in PCa diagnosis.

## Data Availability

The raw data supporting the conclutions of this article will be made available by the authors, without undue reservation.
