# Supplementary material for: Untargeted metabolomics revealed urinary metabolic pattern for discriminating prostate cancer from benign prostatic hyperplasia in Chinese participants
Source: Front Oncol. 2025 Jun 25;15:1604169. doi: 10.3389/fonc.2025.1604169 (PMC12238017; doi:10.3389/fonc.2025.1604169)
Supplement: Supplementary file 1 [file DataSheet1.pdf]

Supplementary Material

1 Supplementary Figures and Tables

1.1 Supplementary Figures

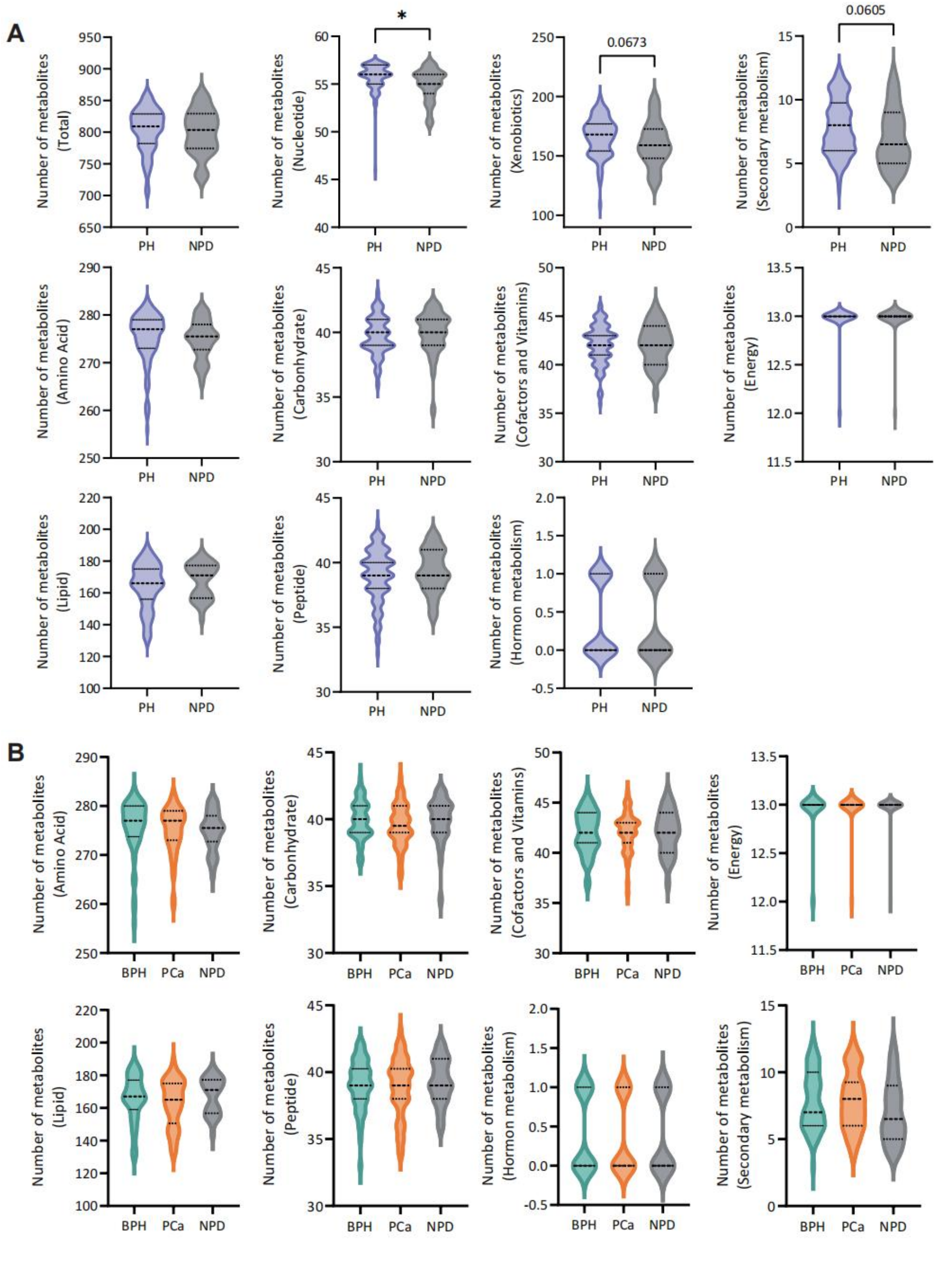

Supplementary Figure 1. Number of detected metabolites in each category from each group. *p < 0.05.

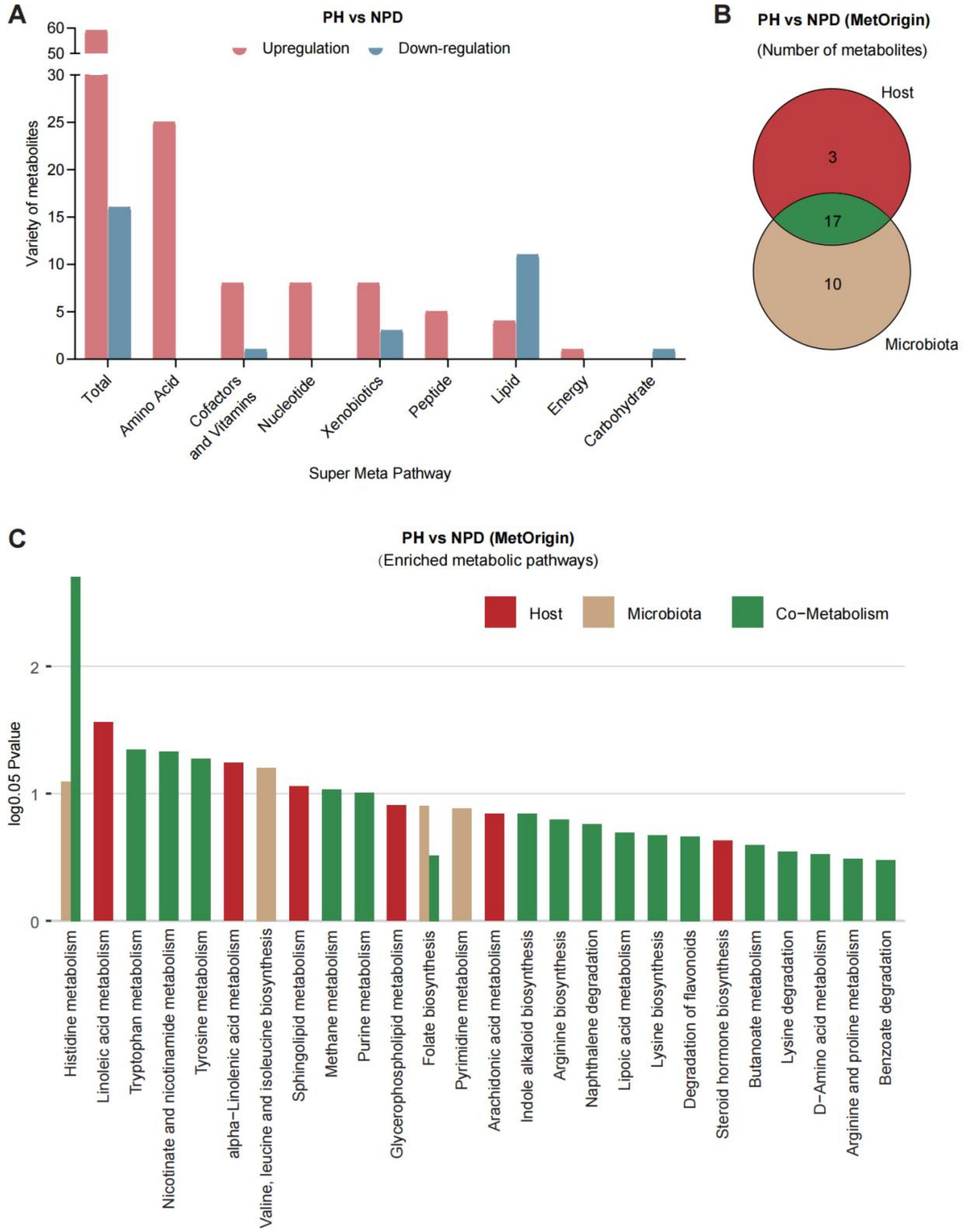

Supplementary Figure 2. Category and origin of differential metabolites between PH and NPD. (A). Bar plot of detected varieties of key metabolites in each category. (B) Venn plot of MetOrigin between PH and NPD. (C). Enriched pathways of MetOrigin between PH and NPD.

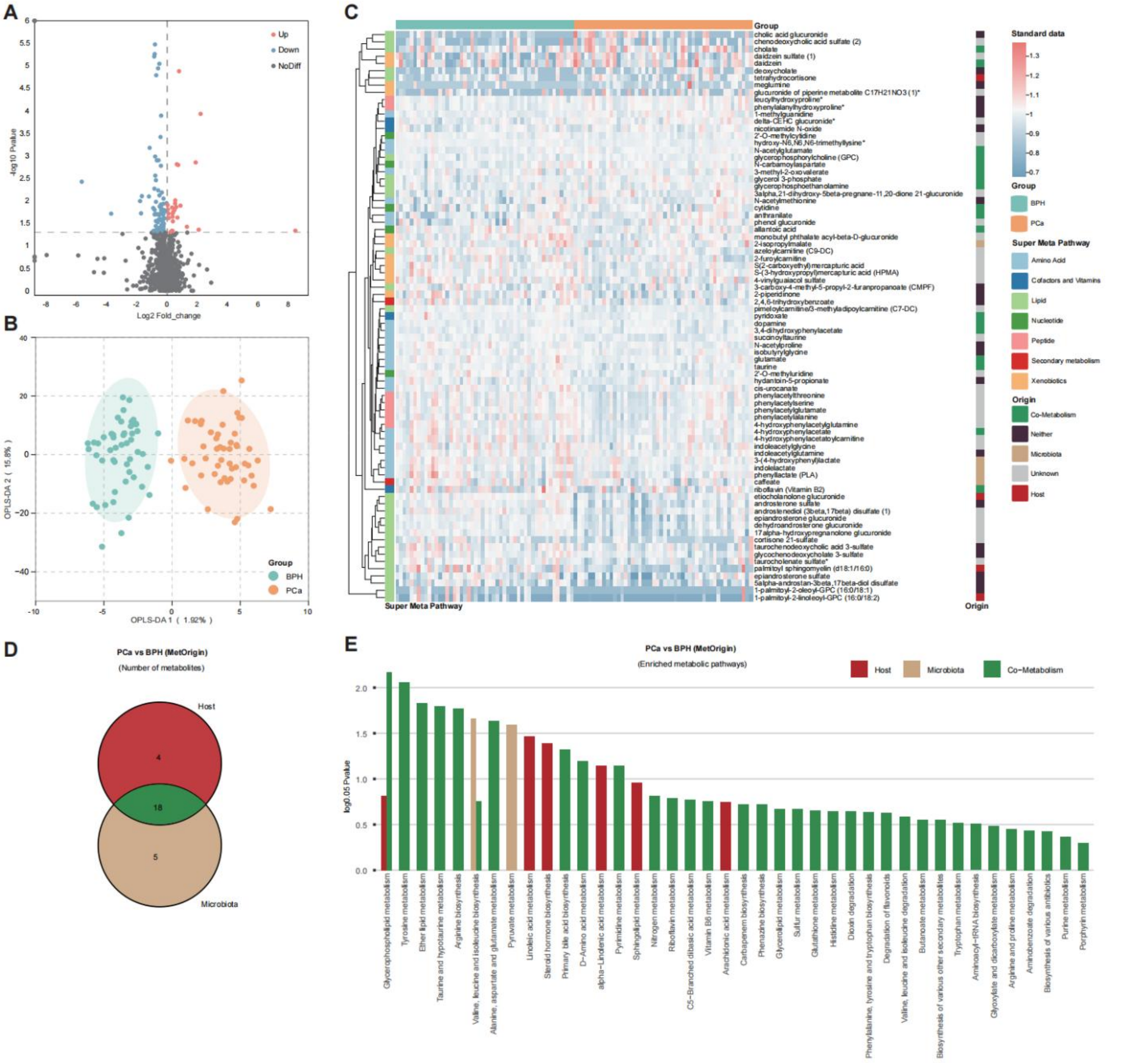

Supplementary Figure 3. Discriminate analysis between PCa and BPH. (A). Volcano plot of changing pattern of metabolites displayed with fold change and p-value. (B). Plot of structural separation after OPLS-DA. (C). Heatmap of 79 differential metabolites (OPLS-DA VIP >1.7). (D). Venn plot of MetOrigin between PCa and BPH. (C). Enriched pathways of MetOrigin between PCa and BPH.

1.2 Supplementary Tables

Supplementary Table 1 List of key metabolites to discriminate PCa from BPH (except those of xenobiotics)

Index	Super Meta Pathway	HMDB ID	VIP	AUC
etiocholanolone glucuronide	Lipid	HMDB0004484	2.59	0.775
2'-O-methyluridine	Nucleotide	HMDB0240328	3.31	0.760
palmitoyl sphingomyelin (d18:1/16:0)	Lipid	HMDB0010169	2.06	0.746
17alpha-hydroxypregnanolone glucuronide	Lipid	/	3.43	0.738
1-palmitoyl-2-oleoyl-GPC (16:0/18:1)	Lipid	HMDB0007972	3.57	0.734
epiandrosterone glucuronide	Lipid	/	2.20	0.715
pimeloylcarnitine/3-methyladipoylcarnitine (C7-DC)	Lipid	/	2.92	0.705
androsterone sulfate	Lipid	HMDB0002759	2.26	0.704
1-palmitoyl-2-linoleoyl-GPC (16:0/18:2)	Lipid	HMDB0007973	2.88	0.704
succinoyltaurine	Amino Acid	/	2.54	0.696
dehydroandrosterone glucuronide	Lipid	/	2.01	0.673
1-methylguanidine	Amino Acid	HMDB0001522	2.50	0.671
N-acetylproline	Amino Acid	HMDB0094701	2.22	0.671
taurochenodeoxycholic acid 3-sulfate	Lipid	HMDB0002486	2.02	0.663
cholic acid glucuronide	Lipid	HMDB0002577	2.20	0.661
allantoic acid	Nucleotide	HMDB0001209	2.15	0.660
taurocholenate sulfate	Lipid	/	2.14	0.652
phenylacetylglutamate	Peptide	HMDB0059772	2.11	0.651
phenylalanylhydroxyproline	Peptide	HMDB0011176	2.10	0.651
pyridoxate	Cofactors and	HMDB0000017	2.88	0.651

Index	Super Meta Pathway	HMDB ID	VIP	AUC
	Vitamins			
2'-O-methylcytidine	Nucleotide	/	1.83	0.650
glycerol 3-phosphate	Lipid	HMDB0000126	2.41	0.648
cortisone 21-sulfate	Lipid	/	2.01	0.645
androstenediol (3beta,17beta) disulfate	Lipid	/	1.88	0.644
leucylhydroxyproline	Peptide	HMDB0028930	2.08	0.643
chenodeoxycholic acid sulfate	Lipid	/	2.20	0.639
3-carboxy-4-methyl-5-propyl-2-furanpropanoate (CMPF)	Lipid	HMDB0061112	1.72	0.638
phenyllactate (PLA)	Amino Acid	HMDB0000779	2.02	0.637
N-acetylglutamate	Amino Acid	HMDB0001138	1.79	0.634
azeloylcarnitine (C9-DC)	Lipid	/	2.15	0.632
4-hydroxyphenylacetate	Amino Acid	HMDB0000020	2.22	0.629
5alpha-androstan-3beta,17beta-diol disulfate	Lipid	HMDB0094682	2.17	0.628
delta-CEHC glucuronide	Cofactors and Vitamins	/	2.12	0.627
phenylacetylalanine	Peptide	/	1.87	0.623
2,4,6-trihydroxybenzoate	Secondary metabolism	HMDB0029649	2.15	0.623
isobutyrylglycine	Amino Acid	HMDB0000730	2.15	0.619
indolelactate	Amino Acid	HMDB0000671	1.81	0.619
hydroxy-N6,N6,N6-trimethyllysine	Amino Acid	/	1.78	0.617
cis-urocanate	Amino Acid	/	1.72	0.616
4-hydroxyphenylacetylglutamine	Peptide	/	1.93	0.614
phenylacetylthreonine	Peptide	/	1.80	0.612

Index	Super Meta Pathway	HMDB ID	VIP	AUC
deoxycholate	Lipid	HMDB0000626	1.72	0.612
taurine	Amino Acid	HMDB0000251	1.74	0.610
glycochenodeoxycholate 3-sulfate	Lipid	HMDB0002497	1.82	0.610
nicotinamide N-oxide	Cofactors and Vitamins	HMDB0002730	2.46	0.608
phenylacetylserine	Peptide	/	1.92	0.606
epiandrosterone sulfate	Lipid	HMDB0062657	2.44	0.602
3alpha,21-dihydroxy-5beta-pregnane-11,20-dione 21-glucuronide	Lipid	/	2.36	0.602
anthranilate	Amino Acid	HMDB0001123	1.85	0.602
cholate	Lipid	HMDB0000619	1.75	0.601
N-carbamoylaspartate	Nucleotide	HMDB0000828	1.96	0.600
4-hydroxyphenylacetatoylcarnitine	Amino Acid	/	2.06	0.600
glutamate	Amino Acid	HMDB0000148	1.75	0.598
indoleacetylglutamine	Amino Acid	HMDB0013240	2.09	0.598
3-(4-hydroxyphenyl)lactate	Amino Acid	HMDB0000755	1.73	0.595
tetrahydrocortisone	Lipid	HMDB0000903	1.73	0.591
glycerophosphoethanolamine	Lipid	HMDB0000114	1.74	0.588
hydantoin-5-propionate	Amino Acid	HMDB0001212	1.70	0.583
indoleacetylglycine	Amino Acid	/	2.05	0.576
caffeate	Secondary metabolism	HMDB0001964	2.15	0.555
phenol glucuronide	Amino Acid	HMDB0060014	1.80	0.555
dopamine	Amino Acid	HMDB0000073	1.71	0.534
riboflavin (Vitamin B2)	Cofactors and	HMDB0000244	1.90	0.531

**Index**	**Super Meta Pathway**	**HMDB ID**	**VIP**	**AUC**
	Vitamins			
3-methyl-2-oxovalerate	Amino Acid	HMDB0000491	2.41	0.515
cytidine	Nucleotide	HMDB0000089	1.90	0.512
N-acetylmethionine	Amino Acid	HMDB0011745	1.91	0.466
glycerophosphorylcholine (GPC)	Lipid	HMDB0000086	1.80	0.465
3,4-dihydroxyphenylacetate	Amino Acid	HMDB0001336	2.02	0.446
